# Human Endogenous Retroviruses as Novel Therapeutic Targets in Neurodegenerative Disorders

**DOI:** 10.3390/vaccines13040415

**Published:** 2025-04-15

**Authors:** Elena Rita Simula, Seyedesomaye Jasemi, Davide Cossu, Milena Fais, Ilaria Cossu, Vanna Chessa, Mattia Canu, Leonardo Antonio Sechi

**Affiliations:** 1 Department of Biomedical Sciences, Division of Microbiology and Virology, University of Sassari, 07100 Sassari, Italy; sjasemi@uniss.it (S.J.); dcossu@uniss.it (D.C.); faismilena@gmail.com (M.F.); i.cossu2@studenti.uniss.it (I.C.); 2 ASL Sassari, SC Anestesia Territoriale Cure Palliatiave, 07100 Sassari, Italy; v.chessa@aslsassari.it (V.C.); mattia.canu@aslsassari.it (M.C.); 3 Struttura Complessa Microbiologia e Virologia, Azienda Ospedaliera Universitaria Sassari, 07100 Sassari, Italy

**Keywords:** HERVs, amyotrophic lateral sclerosis, multiple sclerosis, Alzheimer′s disease, Parkinson′s disease, neurodegeneration, epigenetics, vaccines, monoclonal antibodies

## Abstract

Human Endogenous Retroviruses comprise approximately 8% of the human genome, serving as fragments of ancient retroviral infections. Although they are generally maintained in a silenced state by robust epigenetic mechanisms, specific HERV groups, particularly HERV-W and HERV-K, can become derepressed under specific pathological conditions, thereby contributing to the initiation and progression of neuroinflammatory and neurodegenerative processes. Preclinical studies and clinical trials, such as those investigating monoclonal antibodies, indicate that directly targeting these elements may offer a novel therapeutic strategy. In this review, we provide an overview of HERVs′ biology, examine their role in neurodegenerative diseases such as amyotrophic lateral sclerosis, multiple sclerosis, Alzheimer′s disease, and Parkinson′s disease, and explore their therapeutic prospects, highlighting both the challenges and the potential future research directions needed to translate these approaches into clinical interventions.

## 1. Introduction

The human genome is densely populated by transposable elements, including Human Endogenous Retroviruses (HERVs), which represent the genomic remnants of retroviral infections that occurred millions of years ago in our evolutionary ancestors [1]. These retroviral insertions became permanent within the human genome due to integration events in germline cells, thus being transmitted vertically across generations [2]. Over extensive evolutionary timeframes, most HERV elements have undergone a significant accumulation of mutations, leading to their transcriptional silencing and loss of coding ability [3]. Moreover, cellular epigenetic mechanisms, such as DNA methylation, histone modifications, and RNA interference, efficiently suppress most of these sequences, maintaining genomic stability and preventing potentially harmful gene activation [4].

Despite their generally dormant state, several HERVs retain intact or partially intact regulatory sequences, such as promoters, enhancers, and polyadenylation signals, as well as, in some circumstances, open reading frames (ORFs) capable of generating functional transcripts and proteins [5]. These regulatory sequences allow HERVs to influence gene expression patterns, either through direct transcriptional interference, modulation of neighboring genes, or alteration of chromatin architecture [6]. Emerging evidence underscores the biological significance of these residual elements, revealing that their controlled reactivation plays critical roles in normal physiological processes such as embryonic development, placental function, and immune system modulation [7].

However, the aberrant reactivation of specific HERV families has been increasingly implicated in pathological conditions [8,9], particularly in the context of neurological diseases [10,11,12]. Recent studies have drawn significant attention to the role of HERV-K, especially the HML-2 subtype and HERV-W families, correlating their abnormal activation with enhanced neuroinflammatory responses and progressive neurodegeneration [13,14]. Elevated expression of HERV-K transcripts and proteins has been detected in the brain tissues and cerebrospinal fluid of patients with ALS [15], suggesting a pathogenic contribution through inflammatory cascades and neurotoxicity mediated by the envelope protein [15]. Similarly, the HERV-W-env protein has been prominently observed within the active lesions of patients affected by multiple sclerosis (MS), directly contributing to microglial activation, neuroinflammation, demyelination, and neuronal damage [16,17]. Additional studies have associated increased HERV activation with Alzheimer′s disease and schizophrenia, reinforcing the idea that deregulated HERV expression may constitute a common pathogenic mechanism underlying various neurological disorders [18].

## 2. Biology and Regulation of HERVs

HERVs represent about 8% of the human genome, making them significant genomic elements derived from ancient retroviral infections. In fact, the reference genome assembly includes approximately 450,000 copies per genome, which are classified into nearly 100 families based on shared structural and sequence features [19]. These retroviruses infected ancestral germline cells, permanently integrating their genetic material into the host genome. Subsequently, these integrated retroviral sequences were transmitted vertically across generations following Mendelian inheritance patterns [20]. HERVs have thus become fixed genomic constituents, persisting across evolutionary timescales and becoming an integral component of our genome [21].

The classification of HERVs traditionally relies upon the identity of the transfer RNA (tRNA) molecule used as a primer during reverse transcription. Each HERV family is thus named according to the amino acid corresponding to its associated tRNA, leading to classifications such as HERV-K (lysine), HERV-H (histidine), and others like HERV-W (tryptophan) and HERV-E (glutamic acid) [22]. Additionally, classification schemes also take into consideration the structural characteristics of their long terminal repeats (LTRs), which contain critical regulatory regions responsible for driving transcription and controlling gene expression [23]. These LTRs possess promoter and enhancer activities, making them influential regulatory modules capable of affecting both the HERV elements and neighboring host genes.

**Figure 1:**
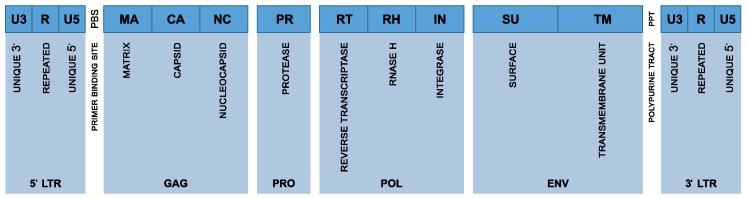
Schematic representation of the HERV proviral genome structure. The figure illustrates the organization of a typical human endogenous retrovirus (HERV) provirus, including the 5′ and 3′ long terminal repeats (LTRs), which are composed of the U3, R, and U5 regions. The internal coding regions are shown in sequential order: gag (encoding MA, CA, and NC), pro (encoding the viral protease, PR), pol (encoding reverse transcriptase, RT, RNase H, RH, and integrase, IN), and env (encoding the surface, SU, and transmembrane, TM, subunits of the envelope protein). Also indicated are the primer binding site, PBS, and the polypurine tract, PPT, which play critical roles in the initiation of reverse transcription and second-strand DNA synthesis, respectively.

Over evolutionary timeframes, most HERV elements have progressively undergone extensive mutational “degeneration” due to genetic drift, insertions, deletions, and point mutations [24]. Consequently, many HERV sequences have lost their capacity for replication and their ability to produce functional viral proteins, as key genes such as gag (capsid protein), pol (polymerase enzymes), and env (envelope proteins) have been disrupted or rendered nonfunctional. A representation of the HERV proviral genome structure is schematized in Figure 1. Despite this widespread genomic decay, certain HERV families, particularly members of the HERV-K family and its HML-2 subtype, maintain relatively intact genomic sequences [25]. These sequences retain the potential to generate transcripts and, under specific physiological or pathological conditions, produce functional viral proteins or even assemble virus-like particles. A genomic analysis identified only 42 HERV loci across the entire human genome containing long viral ORFs, specifically 17 gag, 13 pol, and 29 env sequences. Notably, none of these proviruses were fully intact across all coding regions. Only two HERV-K (HML-2) loci harbored ORFs for all major genes. However, both carried inactivating mutations that preclude any replication competence [26]. The inter-individual variability in HERV content is evident from polymorphic insertions like in HERV-K113 and HERV-K115 sequences, which are present in certain individuals but absent in others, with population-specific differences in their allele frequencies [5]. However, detailed statistical measures, including mean and standard deviation values of non-functional HERVs across individual genomes, are currently lacking. Recent findings show that proviral content varies more extensively among individuals than previously thought, which may influence HERV-related disease susceptibility [27].

Epigenetic regulatory mechanisms exert stringent control over HERV transcriptional activity, predominantly through DNA methylation and histone modifications such as methylation and acetylation. These mechanisms collectively contribute to chromatin remodeling, ensuring that HERV sequences remain transcriptionally silent under normal physiological conditions. Epigenetic silencing prevents the potentially toxic consequences of uncontrolled retroviral expression, including genomic instability and aberrant immune activation [4]. However, a recent study has revealed that environmental and cellular stressors, such as viral infections, oxidative stress, inflammation, and exposure to chemical compounds, can disrupt these epigenetic mechanisms. Such perturbations in chromatin structure or DNA methylation patterns can trigger a selective derepression of HERV sequences, facilitating their aberrant transcriptional activation [28].

In addition to epigenetic control, HERV transcriptional silencing is regulated through interactions with specialized transcription factors and regulatory proteins, notably the KRAB zinc-finger proteins (KRAB-ZFPs) [29,30]. KRAB-ZFPs bind directly to specific HERV loci, recruiting co-repressor complexes that further reinforce transcriptional repression through chromatin compaction and maintenance of repressive histone marks [31]. When these regulatory pathways become compromised due to genetic mutations, a reduced expression of KRAB-ZFPs, or dysregulated cellular signaling cascades, HERV elements can escape repression, resulting in inappropriate transcriptional activation [31].

The aberrant activation of HERV sequences carries significant biological consequences, including the generation of immunogenic viral proteins, such as the envelope protein, and the formation of extracellular virus-like particles. These events can stimulate immune recognition and potentially trigger inflammatory and autoimmune responses.

## 3. Biological Role of HERVs

The biological role of HERVs goes beyond their historically “junk DNA” roles. Recent evidence highlights that HERV-derived sequences contribute significantly to essential physiological processes, particularly during embryonic development and placentation [32]. For instance, syncytin-1 and syncytin-2, envelope proteins encoded by HERV-W and HERV-FRD, respectively, have been co-opted by the human genome to facilitate the cell fusion necessary for the formation of the placental syncytiotrophoblast layer [33]. This layer is critical for maternal–fetal nutrient exchange and immune modulation, ensuring successful pregnancy outcomes. Syncytin not only mediates trophoblast cell fusion but may also actively modulate maternal immune tolerance towards its presence in placental exosomes [34].

Another example are HERV-H elements, which are highly expressed in human embryonic stem cells and are essential for maintaining pluripotency, indicating a regulatory role in early development [35].

Furthermore, HERV sequences actively participate in the regulation of innate immune responses, serving as endogenous sensors of viral infections. These sequences can interact with pattern recognition receptors such as Toll-like receptors (TLRs) and retinoic acid-inducible gene-I-like receptors (RIG-I), initiating antiviral pathways and influencing cytokine production [36]. In addition, certain HERV proteins have been shown to have immunosuppressive properties, suggesting their role in preventing excessive inflammatory responses, which could otherwise lead to autoimmune disorders [37].

HERVs also significantly impact genetic diversity and evolutionary processes. By providing novel regulatory sequences such as promoters, enhancers, and alternative splicing sites, HERV insertions influence host gene expression patterns, contributing to the dynamic regulation of gene networks involved in development, differentiation, and cellular adaptation [38,39,40]. Although significantly reduced in modern humans, their ability to mobilize and induce genetic rearrangements has historically contributed to genomic innovation [41], facilitating speciation events and enabling evolutionary adaptation to changing environmental pressures, highlighting the critical role of HERVs throughout human evolutionary history.

In addition to physiological roles, dysregulated HERV expression has been linked to disease. Some env proteins harbor an immunosuppressive domain that can downregulate immune activation, a property thought to aid viral persistence but which might affect tumor or autoimmune microenvironments [42,43]. Numerous studies have observed HERV upregulation in cancers and autoimmune diseases. For example, HERV-K (HML-2), one of the most biologically intact HERV families, can produce the accessory proteins Rec and Np9 that interact with host signaling pathways (e.g., c-MYC, Notch), suggesting potential oncogenic effects [44]. In myasthenia gravis (MG), an autoimmune disorder characterized primarily by muscle weakness due to antibody-mediated neuromuscular junction disruption, autoantibodies targeting specific epitopes of HERV-K and HERV-W envelope proteins have been identified in patient serum samples [45]. Although myasthenia gravis fundamentally involves autoimmune pathology directed against acetylcholine receptors, the detection of these autoantibodies against HERV epitopes suggests an interplay between HERV activation and autoimmunity and represents novel therapeutic targets.

## 4. HERVs in Neurodegeneration

**Figure 2:**
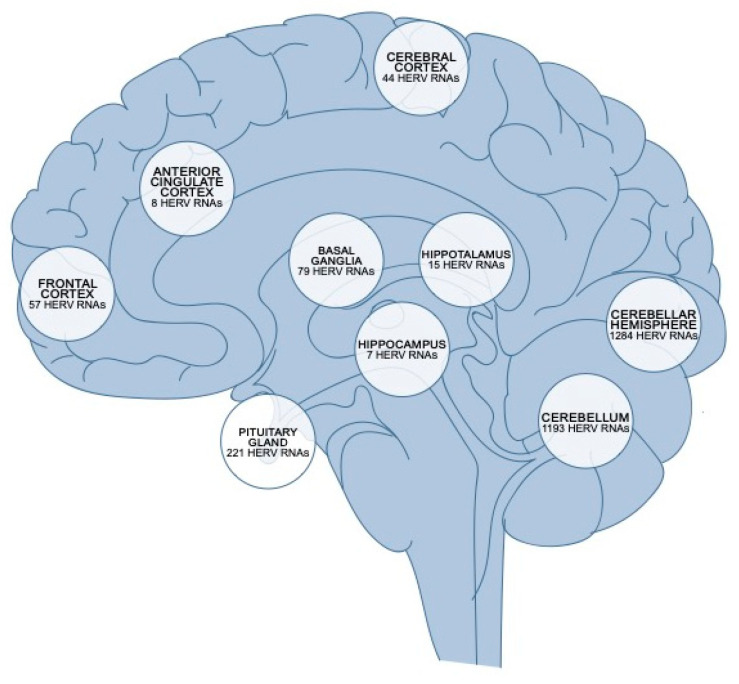
Regional distribution of HERV RNA transcripts in the human brain. Quantification of HERV RNA expression across distinct anatomical regions of the human brain. The cerebellar hemisphere and cerebellum exhibit the highest abundance of HERV RNAs (1284 and 1193 transcripts, respectively), followed by the pituitary gland (221 transcripts) and basal ganglia (79 transcripts). Lower levels are observed in cortical and limbic regions, including the frontal cortex (57), cerebral cortex (44), hypothalamus (15), anterior cingulate cortex (8), and hippocampus (7) [76].

The relationship between HERV reactivation and neurological disorders has become a central topic of investigation, driven by increasing evidence implicating these retroviral elements in neurodegenerative processes. Over the past decade, multiple lines of evidence have illustrated a robust association between the aberrant activation of specific HERV families, notably HERV-K and HERV-W, and the onset or progression of various neurodegenerative diseases. The regional distribution of HERV RNA transcripts in the human brain is represented in Figure 2. The molecular mechanisms underlying these associations are unclear. They may involve intricate interactions between HERV-derived proteins, immune system dysregulation, chronic inflammation, and direct neuronal toxicity, contributing to neuronal injury, synaptic dysfunction, and progressive neurodegeneration [46].

In multiple sclerosis (MS), an inflammatory demyelinating disease of the central nervous system, the elevated expression of transcripts and proteins derived from the HERV-W family has been consistently documented. Specifically, the envelope protein of HERV-W has been prominently identified within active demyelinating lesions [47], where it significantly contributes to the pathogenesis of MS. The HERV-W-env protein acts through the activation of microglial cells and macrophages [48], driving these cells into a pro-inflammatory state characterized by the secretion of cytokines [49] such as interleukin-6 (IL-6), tumor necrosis factor-alpha (TNF-α), and interferon-gamma (IFN-γ). These cytokines amplify the neuroinflammatory cascade, exacerbating oligodendrocyte loss and inhibiting remyelination, ultimately leading to sustained neurological deficits [50]. Clinical studies evaluating the therapeutic strategies targeting HERV-W proteins, such as monoclonal antibodies like temelimab, have demonstrated promising outcomes, including a reduction in neuroinflammation and preservation of neuronal integrity [51]. Besides HERV-W, other endogenous retroviruses (e.g., HERV-K18, HERV-Fc1) have been associated with MS risk or lesions [3,52,53,54]. One of the mechanistic models may be that an initial trigger, such as Epstein–Barr virus infection, recently established as a necessary factor in MS [55], might trans-activate latent HERVs, whose env proteins then sustain chronic CNS inflammation via toll-like receptor signaling and impede remyelination [56]. Not only EBV but also HSV-1 has recently been demonstrated to transactivate HERVs [57].

Amyotrophic lateral sclerosis (ALS), another debilitating neurodegenerative disease characterized by progressive loss of motor neurons, has similarly been linked to aberrant HERV activity, particularly involving the HML-2 subtype of HERV-K [58,59,60]. Different studies have revealed that patients with ALS exhibit increased levels of HERV-K transcripts and proteins within neuronal tissues and biofluids, such as cerebrospinal fluid [15,61]. A transgenic mouse engineered to overexpress neuronal HERV-K env develops progressive paralysis and neurodegeneration, closely mimicking ALS, which provides strong evidence that HERV-K env can be neurotoxic when expressed in vivo [15]. Recent investigations have pointed to the mechanisms by which HML-2 subtype activation contributes to ALS pathogenesis. One notable pathway involves the proteinopathy associated with TAR DNA-binding protein 43 (TDP-43). Elevated expression of HML-2 sequences has been shown to correlate with a significant reduction in the enzyme Asparaginase-like-1 protein (ASRGL1), an enzyme crucial for the removal of abnormal post-translational protein modifications. Deficiencies in ASRGL1 enzyme activity facilitate the aggregation of misfolded TDP-43 proteins, a pathological hallmark of ALS. These TDP-43 aggregates disrupt essential cellular processes, such as RNA metabolism, protein synthesis, and proteostasis mechanisms, ultimately leading to neuronal degeneration and motor neuron death. Consequently, therapeutic approaches aiming to restore the epigenetic silencing of HML-2 or directly inhibit its expression have emerged as potential treatments [62]. Moreover, an intriguing connection has emerged between HERV-K and TDP-43. Aberrant TDP-43 can derepress endogenous retroelements, leading to HERV-K upregulation, and conversely, HERV-K env expression can exacerbate TDP-43 pathology [15,63,64]. Clinically, there are reports indicating that patients with HIV that subsequently developed ALS experienced slowed ALS progression upon antiretroviral therapy, hinting that suppressing HERV activity might be beneficial [65,66,67].

While ALS and MS are the best documented, other neurodegenerative diseases are being investigated for HERVs. Recent studies have indicated their potential involvement in conditions such as Alzheimer′s disease (AD), Parkinson’s disease (PD), and schizophrenia. Sequencing in AD revealed elevated transcripts of multiple HERVs, such as HML4, HARLEQUIN, HERVFC1, HERVK11D, and HERVK11, in AD brains [68]. This may be secondary to chromatin relaxation by the pathogenic tau protein, which can activate transposable elements [69,70]. Genes and HERVs differentially expressed in AD patients compared to controls have been identified [13], showing a widespread genomic distribution of aberrantly expressed HERVs, particularly enriched within the HERV-K superfamily. Several of these HERV insertions exhibit strong correlations with genes whose expression is dysregulated in AD and are frequently located near genes implicated in crucial disease-associated pathways [68].

HERV-K expression is predominantly localized to astrocytes, where it colocalizes with glial fibrillary acidic protein (GFAP), a marker indicative of astrocyte activity. Compared to healthy controls, brain samples from PD patients exhibit a reduced expression of both HERV-K and GFAP. Similarly, decreased HERV-K levels are also evident in the peripheral blood of PD patients, correlating closely with GFAP concentrations. Moreover, lower HERV-K expression correlates with an increased severity [71] and longer duration of PD and with the upregulation of specific miRNAs able to bind the transcript of HERV-K [12]. These findings collectively suggest that HERV-K expression is closely linked to astrocytic functions and disease progression, supporting the hypothesis that HERV-K might play a neuroprotective role in PD [71].

Shifting from the neurodegenerative context, an unsupervised clustering analysis in patients with schizophrenia and bipolar disorder, compared with healthy controls, identified distinct patient subgroups primarily characterized by the presence or absence of HERV-W-env protein antigenemia. Positive HERV-W-env antigenemia was frequently observed among schizophrenia and bipolar disorder patients but rarely detected in healthy controls. The presence of HERV-W-env was associated with elevated inflammatory cytokine levels and increased childhood trauma scores. In schizophrenia, patients positive for HERV-W-env protein exhibited more pronounced maniac symptoms and required higher doses of antipsychotic medication, while bipolar patients with positive antigenemia had an earlier onset of disease. These findings suggest that HERV-W env antigenemia and related inflammatory profiles could serve as biomarkers for stratifying major mood and psychotic disorders into clinically and immunologically distinct subgroups [72].

Finally, it is important to highlight recent evidence supporting the involvement of HERVs in the context of autism spectrum disorder (ASD). Recent studies have documented that specific HERV families exhibit distinctive expression patterns in patients with ASD compared to healthy controls, suggesting their potential utility as biomarkers. Additionally, significantly elevated expression levels of HERV-K and HERV-H env genes have been reported in blood samples from children diagnosed with ASD, indicating a possible association with the pathogenic mechanisms. Furthermore, an abnormal expression of HERV families and cytokines has been observed in children with ASD and their mothers, supporting a correlation between HERV activity and immune activation in ASD [9,73,74,75].

## 5. Therapeutic Strategies Targeting HERVs

The identification of HERV derepression in neurological disorders has spurred the development of several innovative therapeutic approaches, significantly expanding the field of potential interventions targeting neurodegenerative and neuroinflammatory diseases. Current therapeutic approaches can be categorized into three main areas, each addressing different aspects of HERV-related pathology.

Pharmacological interventions aimed at suppressing the transcription and translation of HERV sequences include the utilization of antiretroviral medications, initially developed to treat exogenous retroviruses like HIV, which have shown efficacy in preclinical models by reducing HERV expression and consequently limiting the pathogenic effects of their encoded proteins. Antiretroviral drugs, such as reverse transcriptase inhibitors and integrase inhibitors, have demonstrated the ability to suppress aberrant HERV activation, reduce inflammation, and mitigate neuronal damage in experimental settings [77,78,79].

Targeted immunotherapies directed specifically against HERV-derived proteins have emerged as a highly promising therapeutic strategy. Monoclonal antibodies have been developed to selectively target pathogenic HERV envelope proteins, thereby neutralizing their biological activities and preventing the initiation or exacerbation of inflammatory cascades in a neurological context [80]. Clinical trials using monoclonal antibodies such as temelimab, which specifically targets the HERV-W envelope protein, have shown significant promise in patients with MS, demonstrating reductions in neuroinflammatory markers, protection of myelin integrity, and clinical stabilization [80,81].

Genetic and epigenetic therapies represent an innovative frontier for directly correcting the molecular defects responsible for HERV derepression. These approaches include gene-editing technologies such as CRISPR-Cas9, which can be used to selectively excise or silence active HERV loci at the genomic level preventing their pathogenic activation and expression. Additionally, epigenetic modulation strategies, such as small molecules that enhance DNA methylation or histone modification patterns, are being investigated to restore epigenetic silencing of derepressed HERV sequences [4]. RNA interference (RNAi)-based therapies also hold promise by selectively targeting and degrading pathogenic HERV transcripts inhibiting the production of neurotoxic proteins and interrupting disease-associated pathways. In addition to these main therapeutic categories, complementary approaches that combine multiple modalities to achieve synergistic therapeutic effects are of increasing interest.

## 6. Immunotherapy

Targeting proteins expressed by HERVs, particularly the envelope proteins, represents one of the most promising therapeutic approaches currently under investigation. Monoclonal antibodies directed against specific HERV-derived proteins offer a highly precise means of intervention, given their ability to selectively neutralize the aberrantly expressed viral elements. Temelimab, an antibody specifically designed to target the envelope protein of the HERV-W family, has been extensively studied, particularly in the context of MS. This therapeutic antibody binds to the HERV-W-env protein, effectively neutralizing its pathogenic activity. By blocking HERV-derived envelope proteins, temelimab significantly reduces microglial activation [51,81], a critical initial step in the neuroinflammatory cascade observed in multiple sclerosis and other neurodegenerative disorders. Activated microglia typically produce pro-inflammatory cytokines such as IL-6, TNF-α, and IFN-γ, which perpetuate inflammation, exacerbate demyelination, and contribute to neuronal injury [82]. Consequently, temelimab-mediated neutralization of HERV envelope proteins mitigates the inflammatory response, protecting neuronal integrity and potentially improving neurological function [83]. Data from phase II clinical trials with temelimab have demonstrated encouraging outcomes, including reduced markers of CNS inflammation, decreased lesion formation on MRI scans, and stabilization of clinical symptoms slowing the disease progression and offering neuroprotective benefits.

The concept of targeting HERV envelope proteins through monoclonal antibodies holds considerable potential for application in other neurodegenerative and neuroinflammatory disorders. Conditions such as ALS, AD, and potentially even psychiatric disorders like schizophrenia, in which HERV activation is implicated, could similarly benefit from this therapeutic strategy. Again, the GeNeuro company is presently developing an anti-HERV-K env monoclonal antibody specifically designed for the treatment of ALS (online at: https://patentscope.wipo.int/search/en/detail.jsf?docId=US306969352&tab=NATIONALBIBLIO&_cid=P22-KJQZUJ-45299-1, accessed on 1 March 2025) [56]. Building on the success demonstrated by temelimab in clinical trials for multiple sclerosis, the anti-HERV-K-env monoclonal antibody represents a potential paradigm shift in ALS treatment, specifically addressing pathogenic mechanisms previously untargeted by conventional therapies. Currently, the anti-HERV-K-env mAb developed is advancing through preclinical validation, and its future clinical trials will be critical to determine its therapeutic efficacy, safety profile, and long-term benefits in ALS patients [53].

## 7. Epigenetic Modulation

Under normal conditions, epigenetic regulation is the primary means by which HERVs′ activity is controlled. Our cells treat HERV loci much like other transposable elements, densely packaging them into heterochromatin. DNA methylation of CpG sites in HERV promoters and histone tail modifications create a repressive chromatin environment and represent the two major epigenetic control mechanisms [4,84]. In many HERV families, the LTR promoter is heavily methylated in somatic cells, preventing transcription initiation [85,86]. KAP1 acts as a scaffold to assemble a multi-protein repression complex, including SETDB1 [87,88], which tri-methylates histone H3 on lysine 9, HP1, which binds methylated histones, and the NuRD complex, which contains histone deacetylases (HDACs) [89,90,91]. Additionally, DNA methyltransferases (DNMT1, DNMT3A/B) and methyl-CpG binding proteins collaborate to maintain methylation on HERV sequences across cell division [92]. In embryonic stem cells, HERV repression relies more on histone methylation (since the early embryo globally hypomethylates DNA), but in differentiated cells, DNA methylation becomes crucial [31,44]. Given this, an alternative and increasingly explored therapeutic strategy involves epigenetic modulators to re-establish the proper silencing of these retroviral elements.

A dysregulation of these epigenetic marks can lead to aberrant transcriptional activation of HERV sequences, directly contributing to the pathological states observed in various neurodegenerative and autoimmune diseases. In MS, the hypomethylation of a HERV-Fc1 locus on the X chromosome was reported in patients, aligning with higher HERV-Fc1 RNA levels in the blood. This suggests an epigenetic failure could underlie aberrant HERV expression in MS [3,93]. Another significant example occurs in ALS, where TDP-43 proteinopathy is closely linked to HERV dysregulation. Under physiological conditions, TDP-43 maintains repression of retrotransposons, whereas pathological loss of nuclear TDP-43 in neurons leads to the derepression and accumulation of HERV-K transcripts, potentially exacerbating neurodegeneration [94]. In conditions where HERVs are driving pathology (e.g., MS, ALS), epigenetics might aim to enhance HERV silencing [95].

Histone deacetylase (HDAC) inhibitors represent a promising class of epigenetic drugs for their potential to restore normal chromatin states and suppress pathological HERV expression. By inhibiting HDAC enzymes, these agents induce the hyperacetylation of histone proteins, resulting in a more relaxed chromatin configuration [96]. Paradoxically, although histone acetylation is generally correlated with increased gene expression, studies suggest that HDAC inhibitors may induce compensatory regulatory pathways or influence further repressive histone modifications, facilitating the restoration of epigenetic control over specific HERV loci. Preclinical studies have provided initial evidence that HDAC inhibitors can effectively diminish HERV transcriptional activity, thereby attenuating downstream inflammatory and neurotoxic consequences [97,98,99].

Additionally, demethylating agents such as DNA methyltransferase (DNMT) inhibitors have gained considerable attention due to their capability to modulate DNA methylation patterns directly [100]. Aberrant DNA hypomethylation at promoter regions of specific HERV elements has been consistently correlated with increased expression and pathological activation [101]. Although traditionally, DNMT inhibitors have been developed primarily as anti-cancer therapies, their potential repurposing to re-establish normal DNA methylation landscapes presents a novel therapeutic opportunity. By selectively restoring methylation patterns at critical HERV promoters, DNMT inhibitors may silence aberrantly expressed retroviral sequences, mitigating their pathogenic potential. However, this therapeutic strategy must be carefully tailored to achieve specificity, as global demethylation may inappropriately activate other genomic elements or disrupt normal gene regulation.

Combining epigenetic therapies, such as HDAC or DNMT inhibitors, with monoclonal antibodies designed to neutralize aberrantly expressed HERV-derived proteins could yield synergistic effects. Epigenetic agents could restore proper silencing of HERV loci at the genomic level, significantly reducing their transcriptional activity, while immunotherapeutic interventions would neutralize any residual pathogenic proteins already expressed.

## 8. Vaccine Strategies

Vaccine strategies utilizing HERVs represent an innovative frontier in the fields of neurodegenerative and oncological diseases by exploiting specific immunological and biological features of endogenous retroviral elements. Vaccination approaches aim to selectively exploit the aberrant expression of HERV-derived proteins, such as envelope protein, to trigger targeted immune responses capable of neutralizing the pathogenic effects of these proteins without disrupting essential physiological functions.

An example highlighting the potential of this approach stems from recent studies that have employed strategies based on targeted modulation of the immunosuppressive domain (ISD) of the HERV-W-env protein. Two vaccine formulations utilizing the HERV-W-env protein were compared, one containing the immunosuppressive domain in its wild-type form and another in which the immunosuppressive domain had been modified [102,103]. Data from murine models demonstrated significant differences in immune activation between these formulations. Specifically, the vaccine formulation containing the wild-type ISD induced a robust activation of bone marrow-derived dendritic cells (BMDCs), as evidenced by an elevated expression of costimulatory molecules and pro-inflammatory cytokines. In contrast, the modified immunosuppressive domain formulation showed a diminished capacity to induce such immune responses, highlighting the crucial importance of the precise structural conformation of the ISD in modulating antigen-presenting cell immune activity.

These preclinical findings clearly suggest that manipulating the immunosuppressive domain of the env protein allows for a refined modulation of the immune response, potentially maximizing therapeutic efficacy while minimizing the risk of systemic immunosuppression [102,103].

Another field under exploration uses virus-like particles (VLPs) assembled from HERV proteins. A vaccine based on HERV-K VLPs has been developed, introducing mutations that eliminate infectivity without compromising immunogenicity. These HERV-K VLPs can display env and gag in a virus-sized particle, effectively presenting multiple epitopes to the immune system. Animal studies showed that such VLP vaccines induce both HERV-specific antibodies and T cells, which then slowed the growth of HERV-expressing tumors (e.g., melanomas) in mice [104].

Vaccine strategies targeting HERVs are also relevant to infectious diseases. As mentioned, HIV provides a compelling target for a HERV-based therapeutic vaccine. The idea is to immunize an HIV-infected individual with a HERV antigen (e.g., HERV-K env) to amplify HERV-specific cytotoxic T lymphocyte (CTLs) that can specifically target the HIV reservoir. Since these CTLs would ignore uninfected cells, which do not express HERV-K env, the vaccine-induced response would be selective for infected cells. A study in nonhuman primates demonstrated that a vaccine regimen including HERV peptides could indeed generate T cells that suppressed the simian analog of HIV, SIV, by targeting HERV-expressing cells [105].

These vaccine strategies also hold promise for neurodegenerative and neuroinflammatory diseases. In these pathological contexts, aberrantly activated HERV proteins substantially contribute to persistent inflammatory states and neuronal degeneration. Vaccines designed to minimize intrinsic immunosuppression mediated by ISD, while simultaneously maintaining the capacity to elicit protective immune responses, could offer innovative and highly targeted therapeutic options for diseases such as MS, ALS, and other conditions characterized by HERV-mediated immune dysregulation.

The success of these vaccination strategies will depend on the precise selection of immunogenic epitopes and careful modulation of immune responses to prevent unwanted autoimmune reactions or cross-reactivity that could damage healthy tissues.

An important aspect in developing HERV-targeted therapies is assessing the safety of both the vector and the antigens employed. Baculovirus vector systems, known for their robust antigen expression and high safety profiles, have emerged as valuable platforms for delivering HERV antigens [106]. Recent studies evaluating baculovirus-based DNA vaccines expressing HERV envelope proteins, initially developed for SARS-CoV-2 applications, have provided valuable insights into their safety and tolerability profiles in animal models [107].

## 9. Conclusions

Despite the promising preclinical findings and innovative therapeutic strategies currently being explored, targeting HERVs in the neurodegenerative disorders presents several challenges that must be addressed before the clinical implementation. One of the critical challenges is the complexity of selectively targeting specific HERV sequences within the human genome. Given that HERVs constitute a significant portion of the genome, with numerous closely related sequences dispersed across multiple chromosomes, achieving the necessary specificity to selectively silence or neutralize pathogenic elements, while avoiding the disruption of non-pathogenic or physiologically beneficial HERV sequences, remains a technical challenge.

Additionally, there is still a limited understanding of the precise molecular mechanisms that regulate the activation and silencing of HERVs across different cell types, especially within the complex microenvironment of the central nervous system. The regulation of HERV elements involves different aspects, including epigenetic control, transcription factor interactions, chromatin remodeling, and environmental influences. Another key challenge is the patient-specific variability of HERV activation in different neurological disorders. Factors like genetic makeup, epigenetic modifications, environmental factors, and stages of disease progression can all affect how HERVs are activated in individual patients. This variability highlights the importance of creating personalized diagnostic methods to detect and track HERV activity, along with reliable biomarkers to evaluate treatment effectiveness and anticipate possible side effects or resistance.
